# Practical Observations on Distortions of the Spine, Chest, and Limbs

**Published:** 1823-03-01

**Authors:** 


*Practical Observations on Distortions of the Spine, Chest, and Limbs; together with Remarks on Paralytic and other Diseases connected with impaired or defective Motion.* By William Tilleard Ward, F.L.S. Fellow of the Royal College of Surgeons; of the Medico-Chirurgical Society; and of the Medical Society of London. 8vo. pp. 168. London, November, 1822.

That distortions are amongst the fruits of civilized life, sedentary habits, and indulgence of the appetites and passions, cannot be disputed; for few are the deformities observable among those people who are more nearly in a state of nature. This exemption from many diseases of civilized life seems considerably owing to the bodily labour imposed on them by their natural habits and wants; and the importance of muscular exercise in the preservation of health, has been observed from the remotest antiquity. Nor can we wonder at it. The muscles of the human body outweigh all the other parts, bones, viscera, membranes, and skin, put together. The state of the muscular system is not only influenced by the state of the nervous, vascular, respiratory, and other functions, but powerfully influences them in turn. How much the muscles are influenced by the nerves and blood-vessels, is well known to every person. They owe all their energy and activity to these; and, the moment they are thrown into action, they re-act upon the sensorium and heart, through the intervention of the nerves and blood-vessels. Muscular exercise, therefore, proves an actual stimulus, or excitement to the nervous and vascular systems, and, through them, to the whole of the viscera. The muscles themselves are strengthened by exercise, contrary to all the laws of mechanics in dead machines, and the whole animal economy soon participates in the muscular energy. But it is not merely by the muscular contractions that this excitement is conveyed to the other parts of the system—muscular motion produces a constant succession of *shocks or succussions* throughout the system, that operate not a little in exciting the various functions. This may not be much perceived in a state of health; but, let a febrile or inflammatory condition occur in the whole or part of the frame, and then we see the unequivocal and pernicious effects of muscular action, and *vice versa*. It is fortunate for many, who are deprived of active muscular exertion, that these shocks or succussions result, also, from passive exercise in carriages or on horseback, and thus their health is preserved.

Mr. Ward acknowledges, that the treatment which he here recommends for incurvations of the spine, was first suggested to him by the perusal of a Treatise on Muscular Motion, by Mr. Pugh; and, that the principles have been lately laid down by Mr. Wilson. The particular mode advised by Mr. Wilson (carrying a weight on the head) appears, he thinks, better adapted to the slighter cases of curvature, than to those of great extent or long duration, and may be resorted to as an auxiliary measure, when the spine has nearly recovered its original shape, in order to establish a permanent cure.

Here our author takes occasion to hint his opinion, that many cases of incipient consumption may be connected with that deformity of the chest, in children, commonly called "chicken breast," and proposes the remedy hereafter to be described, (muscular exertion) for the removal of the predisposition.

We must pass over Mr. Ward's first chapter, on the "Influence of Muscular Exercise on the Body," as it consists principally of physiological truths and speculations, which are pretty generally known. We shall, however, give the inferences which he draws, after a full consideration of all the data.

"That the comparative power of muscular parts depends,

"1. On the state of the functions of respiration and circulation, and that increased strength is a consequence of increased vascularity and circulation of blood in the part, and vice versa, a want of tone and power, of a deficient supply of it.

" 2. On the degree of exercise or frequency with which they are called into action.

" 3. On the mental energy or power of volition exerted on them.

" 4. That the most effectual means of encreasing muscular strength is by frequent exercise of the power itself, and, consequently, the preservation of the healthy actions of those functions by which it is influenced.

" 5. That the muscular parts have a constant tendency to contract, by which they adapt themselves to the state of the limb or parts to which they are attached." 15.

Chap. II.—*Curved Spine.* The disease to which our author here confines his attention, does not, he says, affect the bony substance of the vertebræ, (this last being a disease from which he endeavours to distinguish the one now under consideration,) but is confined to the parts connecting them, "and is in its consequences, no less injurious to the general health and happiness of the individual." He defines it "an alteration in the natural form of the spinal column, without caries of its bony structure."

The appearances which our author has met with on dissection, are the following:—the intervertebral substance is generally thinner than natural, but much more on the concave than on the convex side of the curve. In some cases, it has not exceeded more than a third part of its natural thickness. The transversales muscles, inserted into the spinous processes, are elongated, and much finer and smaller on the convex, than on the concave, side of the curve, where they are shorter and fuller. This state is accurately described by Bichat, in his Descriptive Anatomy. "Dans les deviations diverses de l'épine, les muscles suivent la disposition osseuse; ils s'allongent du coté de la convexité, se raccourcissent et se renflent du coté de la concavité. Les faisceaux divers du transversaire épineux m'ont presenté surtout cette disposition." In both instances, the muscles are more pallid than usual; the ligaments, also, are not so strong as in a healthy subject.

This disorder, our author thinks, is on the increase, particularly amongst females in the opulent classes of society—" a circumstance which, perhaps, may be attributed to the present mode of education, in which greater attention is paid, than formerly, to the cultivation of the mind and to female accomplishments, and less time, consequently, allowed for the bodily exercise necessary for the preservation of health." The errors of diet, and the mismanagements during lactation, are, also, to be taken into account.

If the incurvation of the spine take place after six or seven years of age, it appears to him, that a want of proper exercise may be deemed the chief cause.

"The opinions generally entertained upon the subject of distorted spine appear to have been, that it has always had its origin in caries of the vertebræ, or in a morbid state of the bone tending to it. Prior, however, to the occurrence of any alteration in the position of the spinal column, except in those cases where it arises from local injury, we find that there is a considerable decrease of muscular power, and a sense of great general lassitude and weariness: the least bodily exertion induces great fatigue, and the patient, even if permitted, is not inclined to indulge in the sports common to childhood; there is generally derangement of the digestive organs, and an uneasiness which is referred to different parts of the spine. As no particular spot can be pointed out as the seat of disease, these symptoms perhaps are overlooked, till, from the general causes of debility, some part of the muscular structure becomes unable to support the spine in the erect position, and it yields; this perhaps may, in some instances, give rise to unnatural pressure on the parts, and consequently inflammation of the ligaments, absorption of the intervertebral cartilages and caries of the bone; but, that very considerable distortion of the spine, both laterally and anteriorly, may exist for years without such effects being produced, I have had sufficient demonstration, both from the instances of restoration which I have witnessed, and from inspection of the parts in the dead subject." 25.

The *firmness* of cartilage will generally be found in proportion to the *strength* of the muscles. From a number of experiments made by Mr. Wasse, (Philosoph. Trans. vol. xxxiii.) it was ascertained, that there is nearly an inch difference between the height of a body on first rising in the morning, and in the evening. The same writer makes this remark: "all the difference I find between labourers and sedentary people is, that the former are longer in losing their morning height, and sink rather less than the latter."

The greater strength of the intervertebral substance in persons advanced in life, may be assigned as the cause of their exemption from this disorder; Pott having remarked that he had "never seen it at an age beyond forty." Our author thinks, that sufficient importance has not been attached to the influence of the cartilages and ligaments in the production of this disorder. Their relaxation or firmness—increase or decrease of size—power or weakness, will be commensurate with the tone and vigour of the muscular structure. The diminished strength of the ligaments and cartilages is a sequel of muscular debility; and, therefore, by giving power to the muscles, an accession of strength is given to the ligaments and intervertebral substance.

"That to muscular debility we may ascribe the first occurrence of the disease, is confirmed by the method of cure, which, however it may differ as to the particular mode of conducting it, is founded on the principle of giving increased action to those muscles of the spine which have been weakened and extended, and thereby equalising their contractile power with that of their antagonists." 31.

Our author thinks, that spinal distortion, arising from muscular debility, may be distinguished from disease of the bony structure, not only by the mode of its termination, but by the history of the complaint. We shall allow the author to attempt the distinction in his own words.

"In the lateral distortion, the incurvation is commonly gradual and not sudden, and if it occur in the cervical vertebræ there is a second or third curve from the action of the muscles of the spine necessary to preserve the centre of gravity; it is not attended with acute pain, but merely a sense of uneasiness, which may, perhaps, be referred to the fatigue of the muscles connected with the spine. In several cases of long standing that have fallen under my own observation, the patient has never been sensible of any pain or uneasiness in the spinal column or its vicinity, and, except from the alteration in shape, would have been totally unconscious of the approach of the disorder. The length of time which it is in forming is also various, sometimes its progress is slow and insidious, occupying a period of one, two, or three, and in some instances six or seven years or more. Its approaches are for a long time scarcely perceptible, but on the occurrence of any particular disturbance to the constitution, such as febrile indisposition, the spine in the course of one, two, or three months, is found to yield in a greater degree than it had previously done during as many years.

"In the anterior curvature of the spine the curve will also be found very gradual, as it comprehends several of the lower cervical, and the whole of the dorsal and lumbar vertebræ; in some instances it is formed by the dorsal and lumbar only; in these cases likewise the pain is of an obtuse kind, which may probably be referred to the same cause.

"In caries of the bodies of the vertebræ there is a sudden projection of the part; the relative position of the spinous processes is altered, and they are occasionally separated to a greater distance than could be imagined, without a loss of substance anteriorly; in other instances they only approximate more nearly to each other.* The incurvation from within outwards is occasioned by the absorption of the bodies of one or more of the vertebræ. The circumstance of the disease having been preceded by a blow, is, with others, a fair ground for suspicion of caries. The pain, previously to any incurvation taking place from disease of the bone, is more acute than in that arising from weakness, as might be expected from the manner in which inflamma-

---

"* Mr. Copeland relates a case in which the intevertebral substance was removed, and the dorsal vertebræ anchylosed, without there having been any elevation of the bent spinous processes, or distortion of the form of the spine; this case however must be deemed of rare occurrence. —*Copeland's Observations on the Spine, p.* 15."

tion proceeds in parts of a ligamentous, cartilaginous, or bony texture, and it is more confined to the diseased part, and attended with greater febrile indisposition." 36.

Our author considers it a matter of great importance, to distinguish the disease of which he treats from that produced by caries and rickets, "as it is evident, that where any alteration of structure has taken place in the bodies of the vertebræ from the *former*, or where there is a scrophulous disease in the parts, any attempts to cure the distortion by muscular exercise would, by preventing the natural cure by anchylosis, be highly injurious."

*Treatment.*—On the first invasion of the disorder the digestive organs ought to be strictly attended to—the diet should consist of plain animal food once a day, "of which the patient ought to be allowed to eat heartily"—bread and butter, or bread and milk, or tea, for the other meals—passive and active exercise of the spinal and other muscles, as friction, shampooing, percussion, horizontal position, galvanism, &c. —and as active, the excitement of the muscles by volition, or general muscular exercise. These various methods of exciting the warmth of the parts, and promoting a greater flow of blood to them, differ as to effect, only in degree. Their choice and application must be left to the discretion of the practitioner, and they must be regulated by the sensibility of the parts and the state of the disease. The gentler means should be used at first. Shampooing and percussion possess some advantages over friction. These means should be employed for not less than an hour at a time, and repeated twice or thrice in the twenty-four hours—never carrying them the length of exciting pain. As to position, in the anterior curvature of the spine, our author prefers recumbency on the back, although he does not consider it absolutely indispensible. He merely recommends it to be pursued to such an extent as not to be productive of inconvenience to the patient.

"In the lateral incurvation the confinement to a general horizontal posture is all that is requisite, without restricting the patient to any particular position." 43.

The recumbent position, by taking off the superincumbent weight, and thus enabling the parts to regain their former healthy condition, is, our author acknowledges, "a measure of essential importance" in the treatment; and, in many slight cases, may alone be sufficient; but, he thinks, it ought not to be relied on exclusively. When the spine has thus recovered its proper station, the muscles attached to it, and which are its principal support, are left in an atonic state, and incapable of executing their functions. It is obvious that the best means of giving permanency to the cure, and of preventing a recurrence of the disorder, is by giving additional tone and strength to the muscles in question.

"One of the methods that I employ for this purpose and the detail of which will place the subject in the clearest point of view, is the following—a weight appended to a cord is passed over a pulley, and the other extremity, having a strap attached to it, is fastened round the patient's head; the pelvis being fixed, the patient is directed to raise the weight by drawing the head and trunk backwards, and to repeat this effort until fatigue is produced. The frequency of repetition of this exercise of the muscles, and the weight of the body to be raised, must, of course, depend on the patient's strength. After each effort, it is advisable to take rest, by lying down on a couch or sofa, in order that the muscles may not be placed on the stretch and thus prevented from recovering themselves. This mode of exercising the muscles is equally applicable to the anterior curvature of the spine, as to those which take place laterally." 44.

Our author advises a combination of the different means enumerated, as being more efficacious than any one measure however strenuously pursued. He has observed that when the recumbent posture was trusted to alone, a great degree of dyspepsia was often induced, a circumstance that did not occur when action and rest were alternated, which strengthened the digestion as well as the muscular system.

Mr. Ward is no advocate for any mechanical contrivance in correcting the spinal distortion, as he coincides with Mr. Wilson in believing that they injure the bones of the pelvis on which they are made to rest. Seven cases are related illustrative of the methodus medendi above detailed. For these cases we must refer to the work itself.

The second chapter is on Deformity of the Chest. As the bones forming the thorax derive their support from the spinal column, any incurvation of this part will necessarily be accompanied by a corresponding displacement of the ribs and sternum, and the removal of the spinal distortion will usually be followed by an improvement in the form of the chest. But thoracic deformity is not unfrequently found to exist without any derangement of the spine. The general appearance of the chest, in these cases, has procured for it the term chicken-breast.

"It is marked by an apparent projection of the sternum, which seems rather to arise from a loss of the arched form and a flattening of the ribs on each side, than from any unnatural protuberance of the bone itself. Sometimes there is a falling in of the breast bone, producing a preternatural hollow instead of projection of this part of the chest, in which case the edges of the false ribs are frequently turned in upon the lungs, and the ensiform cartilage can scarcely be felt, and not unfrequently one side of the breast is flattened, while there is a corresponding swelling of the opposite side.

"In weakly and delicate children also, independently of any distortion, there is a greater length of chest from the first to the lowest false rib than in the natural state; the clavicles project forwards, as well as the points of the shoulders, and there is not that depth or capacity of chest from the sternum to the spine which may be observed in perfectly healthy individuals; this is particularly apparent when the patient is viewed sideways." 69.

The diminished capacity of the chest is productive of various complaints, as palpitations, dyspnœa, and pulmonic affections. In the treatment our author relies principally on the local means before detailed, with proper attention to diet and the general state of the constitution.

"The method which I have employed with regard to the local means in those cases, where the spine has been exempt from disease, has been that of placing the intercostal muscles and those connected with the anterior part of the chest on the stretch, by placing the patient in a standing position, with the back against a cylindrical piece of wood and the arms extended backwards. By this means an extension of the pectoral muscles is produced, and they are thus brought into full action upon the ribs as well as the muscles of the abdomen which are opponents to them. The position, as well as the condition of the muscles, may be imagined by that of a person in the act of attempting to throw a somerset backwards. While the patient is in this situation he is desired to take deep inspirations. I direct manipulation, and afterwards percussion, to be employed for one or two hours during the day, gradually increasing them in force according to the influence produced on the patient.

"In addition to these means, I usually direct the patient to suspend the body by the arms, and similar modes of exercise, with a view to promote the full action of the pectorales, serrati magni, and postici muscles, &c. on the ribs, to produce the greatest possible extent of elevation of the ribs and sternum, and consequent expansion of the chest." 76.

The 4th chapter is on Contractions of the Limbs. Our author is inclined to view these affections as depending rather on weakness in the extensors than in rigidity or contraction of the flexors of the part bent. The cases of distortion, our author remarks, in which the motion of the limb is not entirely lost, arise generally from inflammation of the joint connected with some internal disorder of the frame, as rheumatism, gout, &c. occasioning deposition of coagulable lymph, or the formation of concretions—at other times, collections of fluid within the cavity of the joint, or abrasion of the cartilages. To these causes may be added spasmodic or paralytic affections, bruises, and other injuries of the muscles, &c. In all these cases, our author thinks, it will be found, upon careful examination, that the muscles of the limb are wasted and flaccid, having almost entirely lost the power of moving the limb from long-continued inaction. The limb is usually bent on the side on which the largest and most powerful muscles are situated. Mr. Ward selects the knee joint for the subject of his observations. It is not easy sometimes to distinguish between complete anchylosis, and a stiffening short of that process, where art may be productive of some benefit. Where inflammation has been so severe and protracted as to produce erosion of the cartilage and a union of the articulating extremities of the bones, it will generally be found, on enquiry, that the pain has been of a very severe and insupportable kind, attended with much watchfulness, the seat of the pain being referred to that part which is immediately under the patella—that the disorder has continued without any diminution of swelling or remission of pain, accompanied with a grating sensation on the slightest motion of the limb—and lastly, that the pain had for some time preceded any appearance of external swelling, the growth of which had been gradual and uninterrupted.

"In those cases where there has been considerable inflammation of the parts, depositions of coagulable lymph, fluid, or gouty concretions in the joint, I think it better to trust their absorption to the influence of gentle exercise of the limb, rather than by the employment of friction, manipulation, or percussion on the joint itself, to incur the risk of the injurious consequences likely to result from the application of local stimulus to parts which, from previous disease, are more liable to the recurrence of inflammatory action. As this use of friction also to the joint itself is commonly attended with some degree of soreness and stiffness of the part on its first application, a circumstance which might mislead the practitioner, and induce him to ascribe it to the effect of disease, I think it better in the first instance to direct friction, manipulation, or percussion to be applied over the extensor muscles of the thigh only. If the angle at which the tibia is fixed on the femur be acute, the patient being placed sitting on a high chair, a line passing over a pulley is affixed to the heel with a small weight attached to it, and he is desired to pull it steadily forwards, and continue to repeat the efforts till fatigue is induced. The first attempts should be continued only for a short time, and in proportion to the increased strength of the extensor muscles; the weight, as well as the length of time occupied in the exercise of the limb, should be gradually augmented. When by a steady perseverance in these means considerable motion has been gained, and sufficient strength acquired to allow the patient to bear his whole weight on the affected limb, a further plan may be adopted of extending the flexor muscles by placing the foot on an inclined plane (which may be made by attaching two pieces of flat board, about one foot and a half in length, and a foot in breadth, to each other, so that when placed on the floor a triangle will be formed, the base of which is the ground, the point of attachment the apex,) the heel resting on the ground, and the toe towards the upper part. In this position the patient should stand on the affected leg only, holding by the back of a chair, so that by advancing the body forwards, or receding, the flexor muscles of the leg may be proportionally extended. This exercise should be persevered in as long as it can be borne without excessive fatigue, and repeated at intervals during the day." 104.

For the cases in illustration we must refer to the work itself.

The 5th chapter is on Paralysis, of which our author presents a pathological and etiological sketch. With the treatment of recent paralytic and apoplectic attacks we need not meddle here, as we have more than once entered pretty deeply into that subject. It is with the chronic effects of the paralysis, the loss or diminution of muscular power, that our business now is. Muscular *exertion* is recommended by our author as the surest means of restoring muscular *power*. It is on this principle he explains the reason why hemiplegiac patients generally recover the use of the *lower* before that of the upper extremities, in consequence of making earlier and more persevering efforts to walk than to work. Friction with the hand, and percussion, appear to have a local effect on the nerves distributed upon the muscles, by increasing their energy, as well as inducing a greater sanguiferous circulation and corresponding increase of strength. "These stimuli I consider inferior in their effect to that excitement produced by the act of volition."

"I have usually advised that the patient should observe a recumbent position, and in that posture make use of muscular exertion till a considerable degree of strength was acquired.

"It has been remarked by patients who have suffered much from the spasmodic twitchings and pains in the night, described by Pott,* that on using considerable muscular exertion, or frequently attempting it, and repeating it at intervals during the day-time, the pains and cramps either did not occur or were lessened. To effect this object it appeared to be necessary to induce complete fatigue." 135.

*Chap.* VI. Chorea Sancti Viti, our author is induced to consider "as a modification of hemiplegia occurring in a young subject." In this we cannot bring ourselves to agree with Mr. Ward. The two principal modes of treatment that

* "Farther remarks on the useless state of the lower limbs, &c."

have been found most successful, viz. purgatives and tonics, do not seem in accordance with Mr. Ward's theory. That it is an affection of the nervous system we have no doubt; but the nature of its cause we cannot but think to be different from that of hemiplegia, which is pressure on the brain or spinal marrow. To Mr. Ward's favourite remedy, muscular exercise, we have not the smallest objection in chorea. He has detailed some cases in which this measure appeared to be serviceable.

The 7th and last chapter contains miscellaneous observations on several subjects. In this chapter our author leads our attention to the good effects of muscular exercise in obstinate cases of chronic rheumatism, combined with the use of mercury. The cure of this disease, in general, he thinks, "may be ascribed exclusively to the administration of that medicine," but several cases have fallen under his care where mercury failed till the aid of muscular exercise was called in.

"The influence of exercise in diminishing the frequency of the pulse is not undeserving of notice in this place. In the case of a young gentleman, whom I directed to use considerable muscular exertion, the first effect was to produce a considerable increased quickness of the pulse; at the expiration of a quarter or half an hour, however, when the immediate acceleration from exercise had abated, the number of beats had been reduced twenty and thirty in a minute. The same effect I have also frequently witnessed in adult age. In a gentleman of forty years of age, whose pulse had regularly, during two years, beat ninety strokes in a minute, it fell to eighty, and subsequently seventy-five, on using daily strong muscular exercise.

"In gouty concretions of the joints, excitement of the muscles, whether by voluntary exercise or other modes, as those of friction, shampooing, or percussion, or a combination of all of them, may be employed with success, observing the caution before given to bring into action the muscles which move the affected joints, and to limit the friction, &c. to those parts only, rather than apply it to the seat of disease." 165.

We have now given a very full account of the little volume before us, and our readers will probably perceive that we are inclined to think favourably of the work. It is written with great modesty and good sense; and it invites our attention to a remedial agent which is much wanted in these days of effeminate manners and sedentary habits.

